# Supplementary material for: Organoids Are Limited in Modeling the Colon Adenoma–Carcinoma Sequence
Source: Cells. 2021 Feb 25;10(3):488. doi: 10.3390/cells10030488 (PMC7996178; doi:10.3390/cells10030488)
Supplement: Supplementary file 1 [file cells-10-00488-s001.zip › Supplementary Table S1.docx]

| Table S1: Gene sets enriched in colorectal cancer tissue (GSE41258) | | | |
| --- | --- | --- | --- |
| NAME | NES | FDR | Category |
| HALLMARK_EPITHELIAL_MESENCHYMAL_  TRANSITION | -2.02 | 0.011 | Development |
| HALLMARK_ANGIOGENESIS | -1.93 | 0.024 | Development |
| HALLMARK_APICAL_JUNCTION | -1.90 | 0.024 | Cellular component |
| HALLMARK_UV_RESPONSE_UP | -1.84 | 0.032 | DNA damage |
| HALLMARK_MYOGENESIS | -1.70 | 0.105 | Development |
| HALLMARK_IL2_STAT5_SIGNALING | -1.62 | 0.170 | Signaling |
| HALLMARK_MITOTIC_SPINDLE | -1.60 | 0.167 | Proliferation |
| HALLMARK_HEDGEHOG_SIGNALING | -1.59 | 0.161 | Signaling |
| HALLMARK_MTORC1_SIGNALING | -1.58 | 0.150 | Signaling |
| HALLMARK_UV_RESPONSE_DN | -1.58 | 0.138 | DNA damage |
| HALLMARK_KRAS_SIGNALING_UP | -1.57 | 0.135 | Signaling |
| HALLMARK_TNFA_SIGNALING_VIA_NFKB | -1.57 | 0.127 | Signaling |
| HALLMARK_G2M_CHECKPOINT | -1.56 | 0.121 | Proliferation |
| HALLMARK_INFLAMMATORY_RESPONSE | -1.55 | 0.119 | Immune |
| HALLMARK_APOPTOSIS | -1.53 | 0.125 | Pathway |
| HALLMARK_COMPLEMENT | -1.52 | 0.130 | Immune |
| HALLMARK_HYPOXIA | -1.52 | 0.124 | Pathway |
| HALLMARK_APICAL_SURFACE | -1.50 | 0.126 | Immune |
| HALLMARK_INTERFERON_GAMMA_RESPONSE | -1.48 | 0.135 | Immune |
| HALLMARK_E2F_TARGETS | -1.47 | 0.133 | Proliferation |
| HALLMARK_COAGULATION | -1.46 | 0.138 | Immune |
| HALLMARK_IL6_JAK_STAT3_SIGNALING | -1.44 | 0.144 | Immune |
| HALLMARK_ALLOGRAFT_REJECTION | -1.43 | 0.145 | Immune |
| HALLMARK_INTERFERON_ALPHA_RESPONSE | -1.42 | 0.150 | Immune |
| HALLMARK_UNFOLDED_PROTEIN_RESPONSE | -1.39 | 0.166 | Pathway |
| HALLMARK_WNT_BETA_CATENIN_SIGNALING | -1.36 | 0.184 | Signaling |
| HALLMARK_TGF_BETA_SIGNALING | -1.30 | 0.234 | Signaling |
| HALLMARK_ESTROGEN_RESPONSE_EARLY | -1.29 | 0.237 | Signaling |
