# Supplementary material for: Organoids Are Limited in Modeling the Colon Adenoma–Carcinoma Sequence
Source: Cells. 2021 Feb 25;10(3):488. doi: 10.3390/cells10030488 (PMC7996178; doi:10.3390/cells10030488)
Supplement: Supplementary file 1 [file cells-10-00488-s001.zip › Supplementary Table S2.docx]

| Table S2: Gene sets enriched in colorectal cancer organoids (GSE57965) | | | |
| --- | --- | --- | --- |
| NAME | NES | FDR | Category |
| HALLMARK_MYC_TARGETS_V2 | -1.91 | 0.002 | Proliferation |
| HALLMARK_UNFOLDED_PROTEIN_RESPONSE | -1.64 | 0.120 | Pathway |
| HALLMARK_UV_RESPONSE_UP | -1.60 | 0.113 | DNA damage |
| HALLMARK_E2F_TARGETS | -1.58 | 0.100 | Proliferation |
| HALLMARK_MYC_TARGETS_V1 | -1.52 | 0.118 | Proliferation |
| HALLMARK_G2M_CHECKPOINT | -1.48 | 0.132 | Proliferation |
| HALLMARK_MTORC1_SIGNALING | -1.38 | 0.197 | Signaling |
