# Supplementary material for: Organoids Are Limited in Modeling the Colon Adenoma–Carcinoma Sequence
Source: Cells. 2021 Feb 25;10(3):488. doi: 10.3390/cells10030488 (PMC7996178; doi:10.3390/cells10030488)
Supplement: Supplementary file 1 [file cells-10-00488-s001.zip › Supplementary_20210209_v04.docx]

**Organoids Are Limited in Modeling the Colon Adenoma–Carcinoma Sequence**

Yoshihisa Tokumaru ^1,2^, Masanori Oshi ^1,3^, Ankit Patel ^1^, Wanqing Tian ^4^, Li Yan ^4^, Nobuhisa Matsuhashi ^2^, Manabu Futamura ^2^, Kazuhiro Yoshida ^2^ and Kazuaki Takabe ^1,3,5–8,^*

^1^ Department of Surgical Oncology, Roswell Park Comprehensive Cancer Center, Buffalo, NY 14263, USA; yoshitoku1090@gmail.com (Y.T.); masanori.oshi@roswellpark.org (M.O.); ankit.patel@roswellpark.org (A.P.)

^2^ Department of Surgical Oncology, Graduate School of Medicine, Gifu University, 1-1 Yanagido, Gifu 501-1194, Japan; nobuhisa@gifu-u.ac.jp (N.M.); mfutamur@gifu-u.ac.jp (M.F.); kyoshida@gifu-u.ac.jp (K.Y.)

^3^ Department of Gastroenterological Surgery, Yokohama City University Graduate School of Medicine, Yokohama 236-0004, Japan

^4^ Department of Biostatistics & Bioinformatics, Roswell Park Comprehensive Cancer Center, Buffalo, NY, 14263, USA; li.yan@roswellpark.org

^5^ Department of Surgery, Niigata University Graduate School of Medical and Dental Sciences, Niigata 951-8510, Japan

^6^ Department of Surgery, University at Buffalo Jacobs School of Medicine and Biomedical Sciences, The State University of New York, Buffalo, NY 14263, USA

^7^ Department of Breast Oncology and Surgery, Tokyo Medical University, 6-7-1 Nishishinjuku, Shinjuku, Tokyo, 160-8402, Japan

^8^ Department of Breast Surgery, Fukushima Medical University School of Medicine, Fukushima 960-1295, Japan

***** Correspondence: kazuaki.takabe@roswellpark.org

**
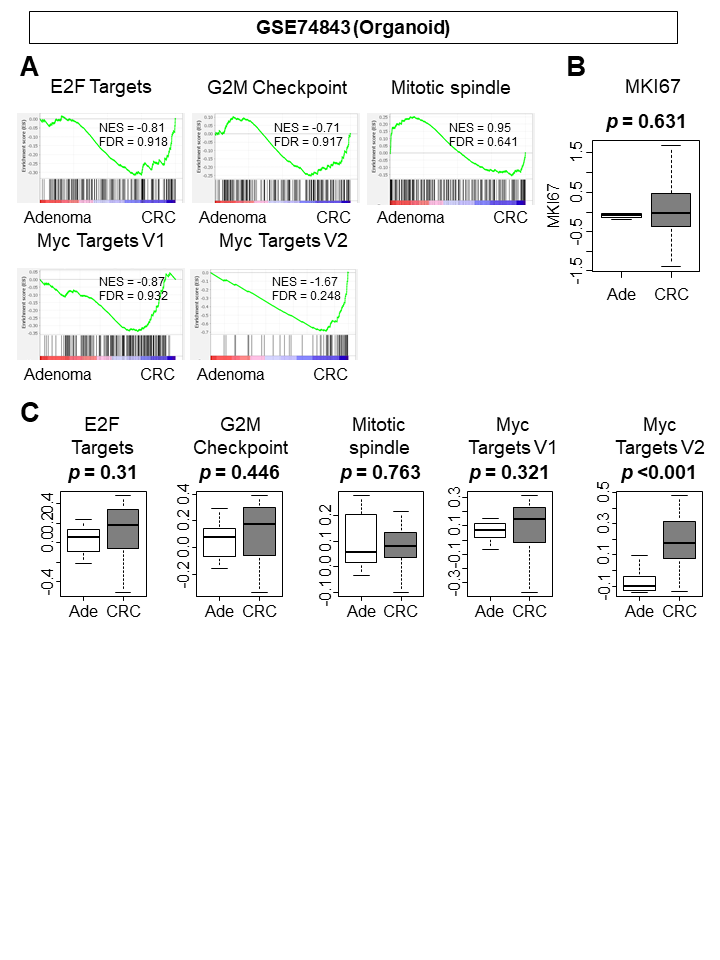
**

**Supplementary Figure S1.** Gene set enrichment analysis (GSEA) of cell proliferation-related gene sets and analysis of MKI67 expression. (A) GSEA of adenoma vs CRC tissue in GSE74843. (B) The MKI67 expression levels in adenoma and CRC in tissue. (C) Single-sample GSEA of GSE74843. Statistical significance was defined as false discovery rate (FDR) < 0.25. Ade, adenoma; CRC, colorectal cancer; FDR, false discovery rate.

**
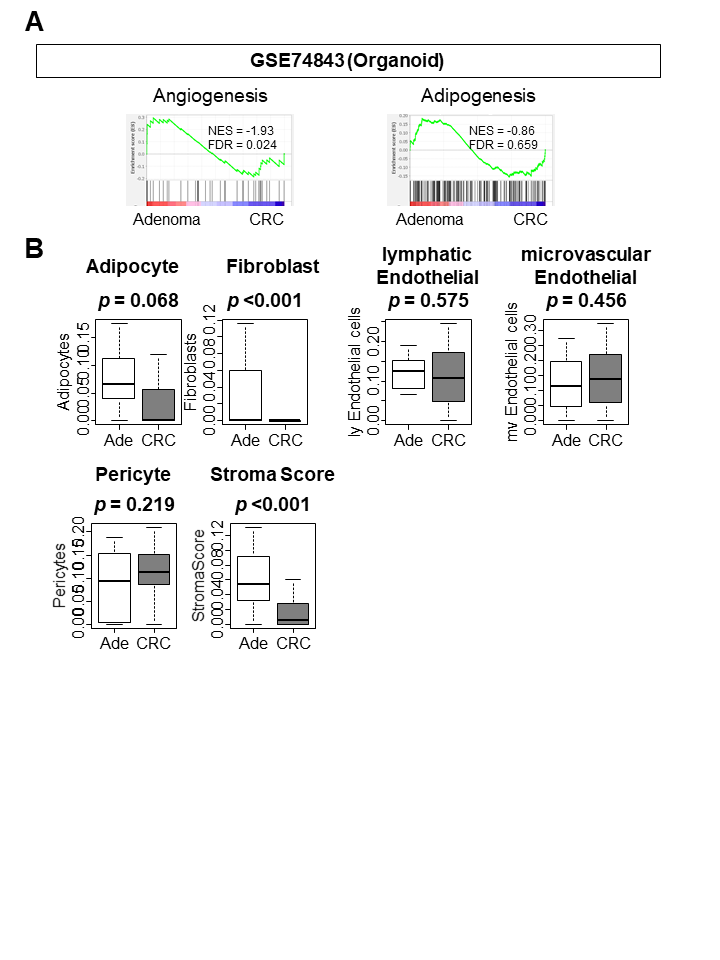
**

**Supplementary Figure S2.** GSEA of tumor immune microenvironment (TME)-related gene sets, the infiltration of stromal cells and the comparison of Stroma Scores. (A) GSEA of tissue sample (GSE74843). (B) Infiltration of stromal cells and comparison of Stroma Scores in organoid samples (GSE74843). Tukey-type boxplots demonstrate the median as well as interquartile level values. Statistical significance was defined as false discovery rate (FDR) < 0.25. Ade, adenoma; CRC, colorectal cancer

**
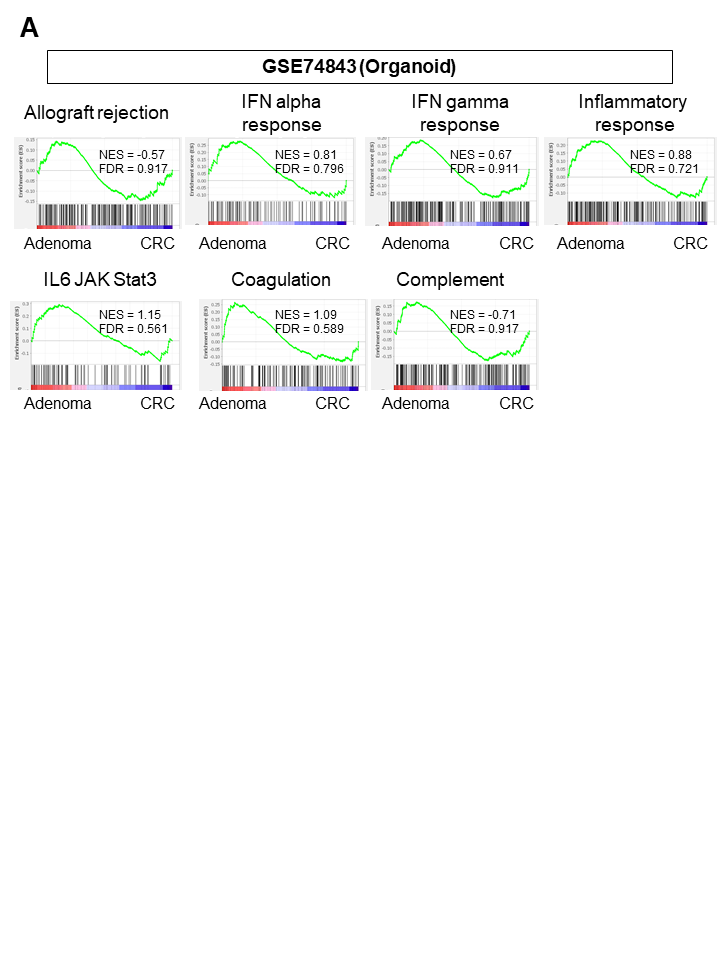
**

**Supplementary Figure S3.** GSEA of gene sets associated with immune response. Analysis of tissue sample (GSE74843).

**
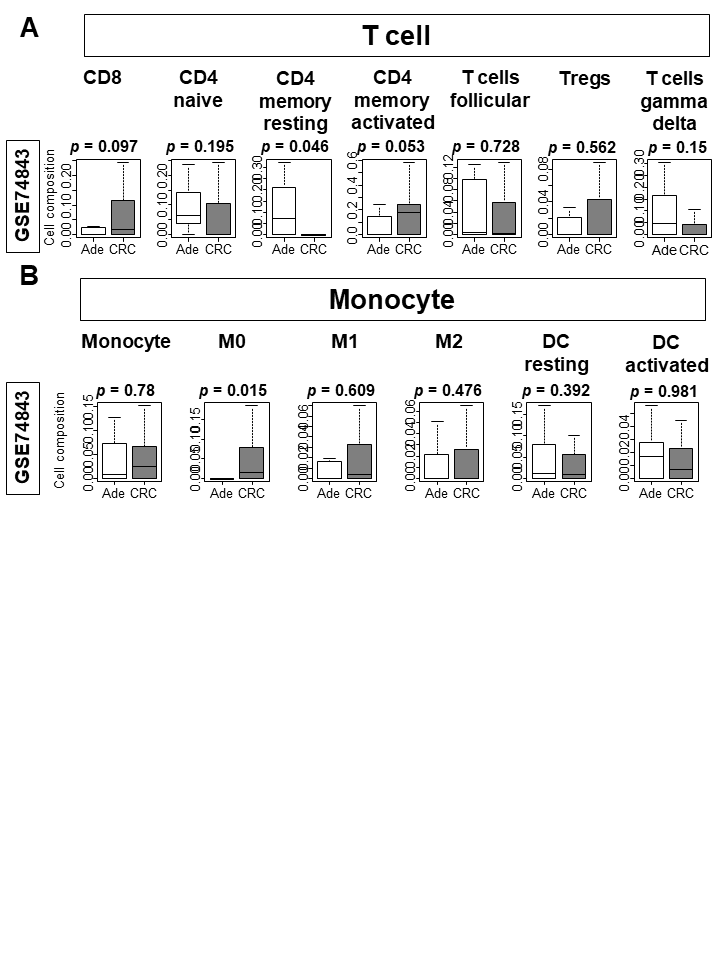
**

**Supplementary Figure S4.** Comparison of the infiltration of immune cells of GSE74843. (A) Analysis of T cells. (B) Analysis of Monocytes. Tukey-type boxplots demonstrate the median as well as interquartile level values. Ade, adenoma; CRC, colorectal cancer

**
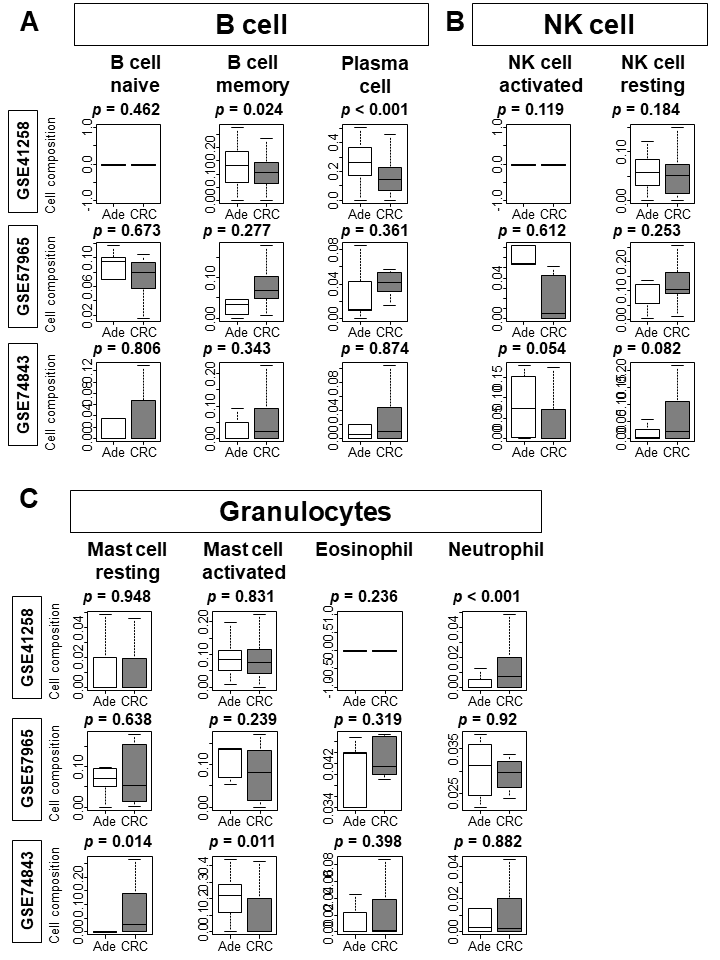
**

**Supplementary Figure S5.** Comparison of the infiltration of immune cells (B cells, NK cells, and Granulocytes). (A) Analysis of B cells. (B) Analysis of NK cells. (C) Analysis of Granulocytes Tukey-type boxplots demonstrate the median as well as interquartile level values. Ade, adenoma; CRC, colorectal cancer; NK cell, natural killer cell

**
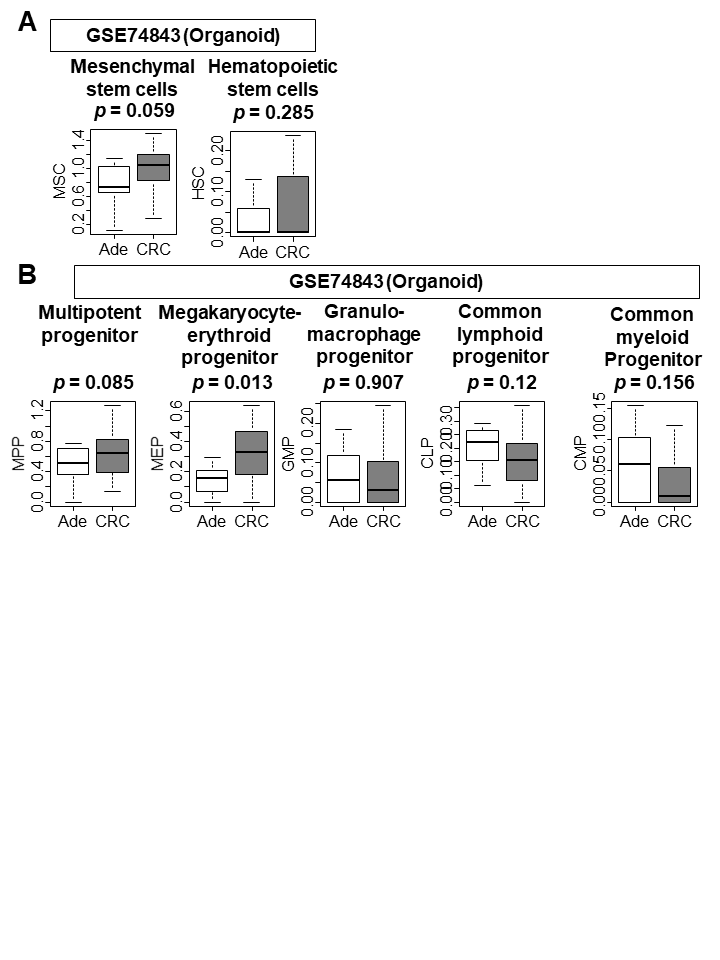
**

**Supplementary Figure S6.** Comparison of infiltration of stem cells and progenitor cells between adenoma and CRC tissue and organoid. (A) Infiltration of stem cells in tissue samples (GSE74843). (B) Infiltration of progenitor cells in tissue samples (GSE74843).
